# Seaweed () Protein Hydrolyzates: A Valuable Source of Short- and Medium-Chain Peptides with Multifunctional Properties

**DOI:** 10.1021/acs.jafc.5c03547

**Published:** 2025-07-17

**Authors:** Enrico Taglioni, Sara Elsa Aita, Carlotta Bollati, Giovanna Boschin, Chiara Cavaliere, Lorenza d’Adduzio, Carmela Maria Montone, Aldo Laganá, Carmen Lammi, Anna Laura Capriotti

**Affiliations:** † Department of Chemistry, 9311 Sapienza University of Rome, Piazzale Aldo Moro 5, Rome 00185 Italy; ‡ Department of Pharmaceutical Sciences, 9304 University of Milan, Via Mangiagalli 25, Milan 20133 Italy

**Keywords:** algae, bioactive peptides, peptidomics, liquid chromatography coupled to high-resolution mass spectrometry, antioxidant properties, dipeptidyl-peptidase IV, angiotensin-converting enzyme inhibitory properties, intestinal trans-epithelial transport, Caco-2 cell

## Abstract

The sustainable valorization of infesting marine biomass offers opportunities to address environmental challenges and emerging nutritional needs. This study investigated the invasive red alga as a potential source of bioactive peptides with antihypertensive and antidiabetic properties. Protein hydrolyzates were generated via enzymatic digestion and fractionated by size exclusion chromatography. Peptidomics analysis using liquid chromatography coupled with high-resolution mass spectrometry identified 362 short-chain and 97 medium-chain peptides. Antioxidant effects were confirmed via diphenyl-2-picrylhydrazyl radical (DPPH), trolox equivalent antioxidant capacity (TEAC), and ferric reducing antioxidant power (FRAP) assays: at 20 mg/mL, short-chain peptides showed a TEAC of 60.8 ± 0.7% and a FRAP activity of 4638.7 ± 87.8%, significantly higher than the medium-chain fraction (36.1 ± 3.6% and 2180.6 ± 25.8%, respectively). Short-chain peptides also demonstrated stronger angiotensin-converting enzyme inhibition (19.53 ± 0.64% at 2.07 mg/mL) compared to medium-chain peptides (12.5 ± 0.42%). Conversely, medium-chain peptides exhibited superior dipeptidyl peptidase IV inhibition. Trans-epithelial transport experiments confirmed bioavailability, with 40 short peptides and 65 medium peptides detected in the basolateral compartment. These findings demonstrate the potential of converting invasive seaweeds into multifunctional ingredients for functional foods or nutraceuticals, supporting marine biotechnology and circular bioeconomy strategies for preventive healthcare and metabolic disease management.

## Graphical abstract

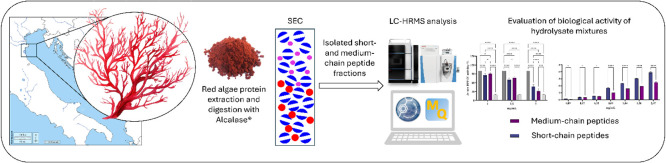

## 1 Introduction

Macrophytic algae, commonly referred to as seaweed, represent a diverse group of marine plants composed of simple cellular structures. They are categorized into green (), brown (), or red algae (), depending on their predominant pigments.^1^ Seaweeds are marine resources with significant potential to fulfill the purpose of the Sustainable Blue Economy and the Bio-Based Circular Economy.^2^ The Blue Economy model extends principles of sustainability and reuse to activities that impact the world’s aquatic ecosystems.^2^ is a red macroalga from the commonly found in coastal waters worldwide.^3^ This species is particularly valued as a natural source of agar, a polysaccharide widely used in sectors such as food production, pharmaceuticals, and biotechnology.^4^ Furthermore, its bioactive components, including antioxidants and antimicrobial agents, highlight its potential in nutraceuticals and biotechnological innovation.^5^ However, uncontrolled growth of this alga in certain areas, leading to algal blooms, has raised concerns regarding its ecological impact on marine habitats and water quality. The increase of anthropogenic impacts worldwide has contributed to the gradual degradation of marine coastal ecosystems, with transitional zones being particularly affected due to significant anthropogenic pressures.^2^ Algal blooms, frequently occurring in the Adriatic Sea, affect human health and produce substantial environmental and economic impacts. These organisms compromise the equilibrium of aquatic ecosystems, for example, by accumulating excessive amounts of wrack onshore. Implementing coordinated efforts to collect and repurpose invasive seaweeds could help mitigate eutrophication by lowering the sea’s nutrient concentrations and the nitrogen-to-phosphorus ratio. Such initiatives align with the European Union’s waste legislation (*Regulation 2008/98/EC*), prioritizing recycling as a sustainable intervention.^6^ Instead of being either composted or used to produce biofuels,^7–9^ the biomass derived from seaweeds is attracting attention due to its high content of bioactive compounds, including soluble dietary fibers, proteins, peptides, minerals, vitamins, polyunsaturated fatty acids, and antioxidants.^3,10^ Edible seaweed species are widely recognized because of their chemical composition.^11,12^ Conversely, less attention has been given to invasive seaweed despite the presence of over 800 macroalgal taxa in Italy.^13^ Recent progress in nutraceutical research and development facilitates the design of patented, proprietary formulations composed of precisely characterized nutraceutical compounds. These formulations address specific health concerns, such as Metabolic Syndrome-related abnormalities, and serve as supportive adjuncts to conventional pharmacological treatments.^14,15^ In this context, considerable attention has been paid to plant-based bioactive peptides, which exert their biological activity due to their high bioavailability. In recent years, short-chain peptides (comprising 2–4 amino acid residues) have gained increasing attention due to their superior absorption rates and enhanced bioactivities compared to longer peptides. These peptides exhibit low cytotoxicity and maintain their biological functions after absorption, as they are resistant to in vivo transformation. They exhibited minimal cytotoxicity and preserved their biological activity after absorption, owing to their resistance to metabolic transformation *in vivo*.^16,17^ The interest in plant-based bioactive peptides also relies on their multifunctional properties to trigger more than one physiological effect through the modulation of diverse targets.^18^ Red algae belonging to the family are desirable due to their high protein content and capacity to generate peptide-rich extracts with promising applications in industrial and biotechnological fields, as well as their documented content of intact proteins and PUFAs, as recently reported by Jiménez-González et al.^19^ Recent studies have demonstrated that protein hydrolyzates derived from thermolysin-mediated hydrolysis of water-soluble proteins (WSP) from the red alga Gracilariopsis chorda effectively inhibit both dipeptidyl peptidase IV (DPP-IV) and angiotensin-converting enzyme (ACE), while also exhibiting significant 2,2-diphenyl-1-picrylhydrazyl (DPPH) radical scavenging activity.^20^ In this context, the present study aims to investigate the potential of as a sustainable source of bioactive peptides, with particular emphasis on identifying and characterizing short- and medium-chain sequences with antihypertensive and antidiabetic properties. The algal samples were chosen to enhance the economic potential of managing seaweed infestations, as significant untapped opportunities remain for their valorization in the nutraceutical and pharmaceutical sectors. This approach supports the circular use of both financial and biological resources. The growing prevalence of chronic metabolic diseases such as hypertension and type 2 diabetes, coupled with the increasing demand for sustainable bioactive ingredients, highlights the need to explore novel, underutilized resources for functional food development. In this context, invasive marine biomass such as offers a dual opportunity: mitigating ecological harm and providing a renewable source of health-promoting compounds. However, unlocking this potential requires overcoming several analytical and biotechnological challenges, including the efficient extraction of proteins from complex algal matrices, the selective generation of bioactive peptides, and the rigorous evaluation of their biological activities and bioavailability. This study addresses these gaps by integrating enzymatic hydrolysis, high-resolution peptidomics, and in vitro bioassays to comprehensively characterize the functionality and absorption potential of short- and medium-chain peptides derived from . Prior research examined the antioxidant activity, bioavailability, and safety of bioactive peptides derived from soybean okara, a byproduct of soy-based food processing. The waste product was reused in this process, emphasizing its health-promoting properties.^21^ Accordingly, size-exclusion chromatography (SEC) was used to isolate short-chain and medium-chain peptide fractions produced from proteins. Then, the purified fractions were investigated by ultrahigh liquid chromatography (UHPLC) coupled with high-resolution mass spectrometry (HRMS) and bioinformatics, specifically tailored for identifying medium and short-chain peptides. Meanwhile, the medium-chain fraction was analyzed using nanoUHPLC-MS/MS. A study based on *in vivo* experiments demonstrated that hydrolyzed produced from proteins inhibited Cholinesterase (ChE).^22^ Therefore, the biological activity associated with the prepared hydrolyzates was investigated for the anti-DPP-IV and antioxidant activities. Afterward, human intestinal Caco-2 cells were used to assess the effects of the hydrolyzates on DPP-IV activity modulation. The bioactivity of food-based peptides relies on their bioavailability at the intestinal level, allowing them to reach the organs where they can exert health-promoting activity intact. In general, the smaller size of peptides has been associated with higher absorption rates than medium- or long-chain ones by enterocytes.^18,23^ Therefore, differentiated Caco-2 cells were employed as a relevant in vitro model to evaluate the transport of peptide mixtures across the intestinal barrier, thereby providing insights into their stability and potential bioavailability in vivo. The rising prevalence of hypertension and type 2 diabetes, combined with the growing demand for sustainable bioactive ingredients, underscores the importance of identifying novel, food-compatible peptides from underutilized marine resources. The valorization of invasive species such as is not only necessary from an ecological and economic standpoint but also presents significant analytical and biotechnological challenges. These include optimizing protein extraction from complex algal matrices, achieving selective peptide generation, and ensuring the functional relevance of the resulting fractions.

## 2 Materials and Methods

### 2.1 Chemicals

All chemicals (reagents and solvents) were from Sigma-Aldrich (St. Louis, MO, USA) unless otherwise stated. Trifluoroacetic acid (TFA) was supplied by Romil Ltd. (Cambridge, UK). Mass grade solvents used for medium-chain peptides were purchased from VWR International (Milan, Italy). Optima LC-MS grade water and acetonitrile (ACN), used for the analysis of short-chain peptides, were purchased from Thermo Fisher Scientific (Waltham, MA, USA).

### 2.2 Samples

 () infesting algae were collected in the northern coastal lagoon of the Adriatic Sea. Sampling sites were selected based on the presence of Gracilaria spp. and the occurrence of algal infestations. The sampling was conducted both onshore (on land) and offshore (in the open sea). Field surveys were conducted from May to June 2024, coinciding with the peak infestation periods. Infested specimens were randomly collected from intertidal and shallow subtidal zones at each site using handpicking and wading techniques. A minimum of 10 samples were collected per site to ensure representative sampling. Collected samples were placed in labeled zip-lock plastic bags containing seawater to prevent desiccation. Samples were transported to the laboratory in an icebox at 4 °C to minimize degradation. Upon arrival, samples were gently rinsed with filtered seawater to remove debris and epiphytes. Samples were freeze-dried by a Heto PowerDry LL1500 (Thermo Fisher), finely ground in a mortar, and stored at −20 °C until use.

### 2.3 Protein Extraction and Digestion

A total of 6 g of freeze-dried macroalgae was divided into 12 Falcon tubes (15 mL each) in aliquots of 500 mg each. Each aliquot underwent extraction using 10 mL of buffer solution containing 50 mmol/L Tris-HCl at pH 8.5 and 2% (w/v) sodium deoxycholate (SDC). The samples were incubated on ice for 1 h, with intermittent vortexing for 1 min every 15 min, and then sonicated in an ultrasonic bath for an additional hour. Insoluble debris was removed by centrifugation at 20,000 × *g* for 10 min at room temperature. Protein content in the supernatant was determined using the Bicinchoninic Acid (BCA) Assay, with bovine serum albumin as the standard, previously described,^24^ resulting in a protein concentration of 1.64 mg/mL. After quantification, the freeze-dried microalgae samples were subjected to hydrolysis with Alcalase added at a 1:10 enzyme-to-protein ratio and incubated at 60 °C for 4 h, as previously optimized.^25^ The reaction was stopped by acidifying the mixture to pH 2 with trifluoroacetic acid (TFA). Samples were then centrifuged at 20,000 × *g* for 10 min at room temperature to eliminate SDC, an acid-insoluble detergent. Finally, the supernatants from each aliquot were pooled together, and the volume was reduced to approximately 1.4 mL to reduce the number of chromatographic runs described below to 14 (100 μL per injection).

### 2.4 Peptide Separation

The Alcalase hydrolyzate was fractionated by size exclusion chromatography (SEC) using a BIOBASIC SEC 120 column (5 μm, 150 × 7.8 mm, Thermo, Waltham, MA, USA) coupled to a Shimadzu Prominence LC-20A system, comprising a CBM-20A controller, two LC-20AP preparative pumps, and a DGU-20A3R online degasser. Peptide detection was carried out at 214 nm using an SPD-20A UV detector equipped with a 10 mm, 12 μL preparative cell operating at a maximum pressure of 12 MPa (1750 bar). A Shimadzu FRC-10A autocollector was used for fraction collection. Chromatographic data were processed with LabSolution version 5.53 (Shimadzu, Kyoto, Japan). Peptides were eluted isocratically at 1 mL/min with H_2_O/0.1% TFA (v/v) as the mobile phase. Two fractions were collected: medium-chain peptides (1–5 min) and short-chain peptides (6–10 min). Afterward, the fractions containing short-chain peptides were combined. A small aliquot corresponding to approximately 1 mg of digested proteins was dried and reconstituted in 100 μL of H_2_O for the analysis described in Section 2.4 The remaining portion was dried and subjected to subsequent biological assays. The dry weight of the mixture was 508 mg (8.5%, *w/w*). Similarly, an aliquot of the pooled fractions containing medium-chain peptides, with the same concentration as the previous one, was purified using solid-phase extraction (SPE), as detailed below for the analysis described in Section 2.5. The remaining portion was dried and subjected to subsequent biological assays. The dry weight of the mixture was 330 mg (5.5%, *w/w*).

#### 2.4.1 Purification of Medium-Chain Peptides

Bond Elut C18 EWP cartridges (50 mg) were first conditioned with 3 mL of acetonitrile (ACN), followed by 3 mL of water containing 0.1% trifluoroacetic acid (TFA). After loading the microalgae extract onto the cartridges, they were washed with 3 mL of 0.1% TFA in water. Peptides were then eluted using 500 μL of a 50:50 (v/v) ACN/H_2_O solution containing 0.1% TFA. The combined eluates were dried under vacuum using a SpeedVac SC250 Express (Thermo Savant, Holbrook, NY, USA), and the dried samples were reconstituted in 100 μL of 0.1% formic acid in water.

### 2.5 Short-Chain Peptide Analysis by Ultrahigh Performance Liquid chromatography-MS/MS

As previously described, the short-chain peptide mixture was analyzed using UHPLC-HRMS in a suspect screening mode.^26^ The system consisted of a Vanquish binary pump coupled to a hybrid quadrupole-Orbitrap Q Exactive mass spectrometer (Thermo Fisher Scientific) with a heated electrospray ionization source (ESI). The ESI source was operated in positive mode and set up as previously reported.^27^ Peptide separation was performed on a Kinetex XB-C18 column (100 × 2.1 mm, 2.6 μm particle size, Phenomenex, Torrance, CA, USA) maintained at 40 °C. Spectra were acquired in positive ion mode over an *m*/*z* range of 150–750 with a resolution of 70,000 (full width at half-maximum, fwhm) at *m*/*z* 200 using an inclusion list with the computationally derived *m*/*z* of short peptides. MS/MS spectra were acquired in top 5 data-dependent acquisition (DDA) at 35% higher-energy collisional dissociation (HCD) and resolution of 35,000 (fwhm at *m*/*z* 200). All analyses were conducted in triplicate. For the identification of the short endogenous peptidome, a customized data processing workflow, developed by our research group and implemented in Compound Discoverer 3.1 (Thermo Fisher Scientific),^26^ was employed. This workflow facilitated the extraction of *m*/*z* values from raw data, alignment of chromatographic runs, and removal of signals originating from blanks or masses lacking MS/MS spectra. Furthermore, it enabled filtering of features not included in the predefined mass list used for short peptide acquisition. Short peptide sequences were identified through manual interpretation of MS/MS spectra, supported by comparison with *in silico*-generated spectra generated by mMass.^28^

### 2.6 Medium-Chain Peptide Analysis by Nanohigh-Performance Liquid Chromatography-MS/MS

Medium-sized peptides were analyzed by nanoHPLC-MS/MS as previously reported.^29^ Analyses were performed using an Ultimate 3000 nanoHPLC system (Thermo Fisher Scientific, Bremen, Germany) coupled to a hybrid linear trap-Orbitrap Elite mass spectrometer (Thermo Fisher Scientific). The mass spectrometer was calibrated weekly using the Pierce LTQ Velos ESI Positive Ion Calibration Solution, following the manufacturer’s guidelines. The mass accuracy was kept below 1.5 ppm without the use of lock-mass correction. For chromatographic separation, 20 μL of each sample was injected. The medium-chain peptide mixture was analyzed as previously described,^30^ with some modifications. A dual-pump configuration was employed for the analysis. Samples were initially preconcentrated on an Acclaim PepMap 100 C18 μ-column (300 μm i.d. × 5 mm; Thermo Scientific), followed by separation on an EASY-Spray analytical column (15 cm × 75 μm i.d., PepMap C18, 2 μm particle size, 100 Å pore size; Thermo Scientific) operated at 300 nL/min and maintained at 35 °C. Chromatographic separation was achieved using a 100 min multistep gradient with H_2_O/HCOOH 99.9:0.1 (phase A) and ACN/HCOOH 99.9:0.1 (phase B): 1% B for 5 min, 1–5% B in 2 min, and 5–35% B in 90 min. Later, a 10 min washing step with 90% B and a 30 min re-equilibration step at 1% B were carried out. Full-scan MS spectra were acquired at *m*/*z* 300–2000 mass range at 30,000 (fwhm at *m*/*z* 400) resolution. MS/MS spectra were obtained in top 10 data-dependent acquisition mode at a resolution of 15,000 (fwhm) using higher-energy collisional dissociation (HCD) with a normalized collision energy of 35%, an isolation window of 2 *m*/*z*, and dynamic exclusion settings. Singly charged and unassigned precursor ions were excluded. Each sample was analyzed in triplicate. Xcalibur software (version 2.2 SP1.48, Thermo Fisher Scientific) was used for data acquisition, and MS spectra were searched against the UniProt protein sequence database of the *Gracilaria* genus (taxonomy ID 2774, comprising 3670 entries), downloaded on November 9, 2024. MaxQuant (v1.6.3.4)^31^ using unspecific digestion, no fixed modification, and variable modification for oxidation of methionine and acetylation of protein N-termini. The minimum peptide length was set to 5 amino acids. Protein identifications were accepted if they included at least one unique razor peptide for the protein group. The false discovery rate was at 0.01 for peptide and protein identifications. Reverse and contaminant hits were removed manually.

### 2.7 Cell Cultures

Caco-2 cells, obtained from INSERM (Paris, France), were routinely maintained and subcultured according to a previously optimized protocol.^32^ Cells were maintained at 37 °C in a humidified atmosphere composed of 90% air and 10% CO_2_, using DMEM supplemented with 25 mM glucose, 4 mM stable l-glutamine, 3.7 g/L NaHCO_2_, 1% nonessential amino acids, 100 U/mL penicillin, and 100 μg/mL streptomycin, along with 10% heat-inactivated fetal bovine serum (FBS).

#### 2.7.1 3-(4,5-Dimethylthiazol-2-yl)-2,5-diphenyltetrazolium Bromide (MTT) Assay

Caco-2 cells at a density of 30,000 cells per well in 96-well plates were treated with short- and medium-chain peptides at concentrations of 0.1, 0.5, 1.0, and 5.0 mg/mL, or with the vehicle (H_2_O), in complete growth medium. Treatments were carried out for 48 h at 37 °C in a 5% CO_2_ atmosphere, following established protocol following ref 32.

### 2.8 Direct Antioxidant Activity of Short- and Medium-Chain Peptides

#### 2.8.1 Diphenyl-2-picrylhydrazyl Radical (DPPH) Assay

The DPPH assay was carried out following a standard protocol with minor modifications. In brief, 45 μL of a 0.0125 mM DPPH methanol solution was added to 15 μL of short- and medium-chain peptide solutions at final concentrations of 1.0, 5.0, 10.0, and 20.0 mg/mL and incubated for 30 min at room temperature in the dark. The absorbance was measured at 520 nm to evaluate radical scavenging activity

#### 2.8.2 2,2′-Azino-bis­(3-ethylbenzothiazoline-6-sulfonic) Acid Diammonium (ABTS) Salt Assay

The TEAC assay is based on the ability of antioxidants to reduce the ABTS+• radical, which was generated by reacting a 7 mM ABTS solution (Sigma-Aldrich, Milan, Italy) with 2.45 mM potassium persulfate in a 1:1 ratio. The mixture was then kept in the dark at room temperature for 16 h. For the assay, 10 μL of short- and medium-chain peptide solutions (either at 5.0, 10.0, or 20.0 mg/mL) were added to 140 μL of diluted ABTS+• and incubated for 30 min at 30 °C. The absorbance was read at 730 nm using a microplate reader (Synergy H1, Biotek), and the TEAC values were calculated using a Trolox (Sigma-Aldrich, Milan, Italy) calibration curve (60–320 μM).

#### 2.8.3 Ferric Reducing Antioxidant Power (FRAP) Assay

The FRAP assay assesses a sample’s capacity to reduce ferric ions (Fe^3+^) to ferrous ions (Fe^2+^). For the assay, 10 μL of short- and medium-chain peptide solutions at final concentrations of 1.0, 5.0, 10.0, and 20.0 mg/mL were mixed with 140 μL of freshly prepared FRAP reagent, i.e., 1.3 mL of 10 mM TPTZ (Sigma-Aldrich, Milan, Italy) in 40 mM HCl, 1.3 mL of 20 mM FeCl_3_·6H_2_O, and 13 mL of 0.3 M acetate buffer (pH 3.6). The reaction mixtures were incubated at 37 °C for 30 min, and absorbance was subsequently measured at 595 nm using a Synergy H1 microplate reader (Biotek).

### 2.9 Evaluation of the Potential Hypoglycemic Activity of Short- and Medium-Chain Peptides

#### 2.9.1 *In Vitro* Measurement of the DPP-IV Inhibitory Activity

Each reaction mixture (50.0 μL) was initially prepared in microcentrifuge tubes by combining 30.0 μL of assay buffer (20 mM Tris-HCl, pH 8.0, containing 100 mM NaCl and 1 mM EDTA), 10.0 μL of peptide samples (at either 1.0, 2.5, or 5.0 mg/mL) or sitagliptin at 1.0 μM (as a positive control), and 10.0 μL of purified human recombinant DPP-IV enzyme. The reaction mixtures were then transferred to a 96-well solid plate, and the reactions were initiated by the addition of 50.0 μL of substrate solution (5 mM H-Gly-Pro-AMC). The plate was incubated at 37 °C for 30 min, after which fluorescence was measured using a Synergy H1 microplate reader (BioTek) at 360/465 nm.

#### 2.9.2 *In Vivo* Measurement of the DPP-IV Inhibitory Activity

Caco-2 cells at a density of 30,000 cells were seeded in black 96-well plates with clear bottoms. Two days postseeding, the spent medium was removed, and cells were treated with 100 μL per well of short- and medium-chain peptides (at either 2.5, 5.0, or 10.0 mg/mL) or with vehicle control (C), in growth medium for 6 h at 37 °C. Following treatment, the medium was discarded, and cells were washed once with 100 μL of PBS lacking Ca^2+^ and Mg^2+^. Subsequently, 100 μL of Gly-Pro-AMC substrate (50.0 μM in PBS) was added. Fluorescence resulting from AMC release was measured every minute for 10 min using a Synergy H1 fluorescence microplate reader at 360/465 nm.

### 2.10 Evaluation of the Potential Antihypertensive Activity of Short- and Medium-Chain Peptides

The ACE inhibitory activity assessment was conducted *in vitro* by measuring the formation of hippuric acid (HA) from hippuryl-histidyl-leucine (HHL), a synthetic substrate that mimics angiotensin I. The experimental procedure followed previously described protocols.^33,34^ Samples (2.5 mM HHL and the peptide mixture, in 100 mM Tris-HCOOH, 300 mM NaCl, and 10 μM ZnCl_2_, pH 8.3) were preincubated at 37 °C for 15 min, after which 15 μL of ACE solution was added. After 60 min at 37 °C, the reaction was stopped and extracted twice with 600 μL of ethyl acetate. The organic solvent was then evaporated and analyzed by HPLC using an Agilent 1200 Series system (Agilent Technologies, Santa Clara, USA) equipped with a Lichrospher 100 C18 column (4.6 × 250 mm, 5 μm; Grace, Italy) to measure the peak areas of HA.

### 2.11 Caco-2 Cell Culture and Differentiation

A modified version of a previously used technique was employed to cultivate Caco-2 cells.^35^ Cells were seeded in the apical (AP) and basolateral (BL) compartments of a Transwell system for 2 days at a density of 3.5 × 105 cells/cm^2^ in a complete medium with 10% FBS. A 10% FBS medium was added to the AP and BL compartments after 2 days. The differentiation of cells happened in 18–21 days, with three weekly changes to 10% FBS medium in between. The transepithelial electrical resistance (TEER) of the differentiated cells was measured with a Millicell voltmeter (Millipore Co., Billerica, MA, USA) to monitor the integrity of the cell monolayers.

### 2.12 Trans-Epithelial Transport of Peptide Hydrolyzates

The integrity and differentiation of the cell monolayer were verified by TEER measurement before trans-epithelial transport testing. Using previously reported circumstances,^35^ the trans-epithelial transport of short- and medium-chain peptides was evaluated using differentiated Caco-2 cells in a transport buffer containing 137 mM NaCl, 5.36 mM KCl, 1.26 mM CaCl_2_, 1.1 mM MgCl_2_, and 5.5 mM glucose. To mimic the physiological pH conditions of the small intestinal mucosa, the apical (AP) solution was adjusted to pH 6.0 using 10 mM morpholinoethanesulfonic acid, while the basolateral (BL) solution was maintained at pH 7.4 using 10 mM *N*-2-hydroxyethylpiperazine-N-4-butanesulfonic acid. Before initiating the transport assay, the cells were equilibrated in HBSS at 37 °C for 15 min. For the transport experiment, the AP compartment received 500 μL of the transport buffer containing short- and medium-chain peptides at a final concentration of 0.5 mg/mL, whereas the BL compartment was filled with 700 μL of buffer. AP and BL solutions were collected post-2-h incubation at 37 °C and stored at −80 °C for subsequent analysis. All trans-epithelial transport experiments were performed in duplicate.

### 2.13 Statistical Analysis

Data are presented as mean ± standard deviation (SD). Statistical significance was defined as a p-value <0.05. One-way and two-way ANOVA were used for statistical analysis, followed by Dunnett’s and Tukey’s posthoc tests, as appropriate (GraphPad Prism 9, GraphPad Software, La Jolla, CA, USA). The Venn diagram was generated using Venny 2.1.0 (https://bioinfogp.cnb.csic.es/tools/venny/index.html).

## 3 Results

### 3.1 Effects of the Short- and Medium-Chain Peptides on Cell Vitality

MTT assays were performed to identify hydrolyzate concentrations that could potentially exert cytotoxic effects on Caco-2 cells. Notably, no cytotoxicity was observed at concentrations up to 10.0 mg/mL following 24 h of treatment (Figure 1).

**Figure 1:**
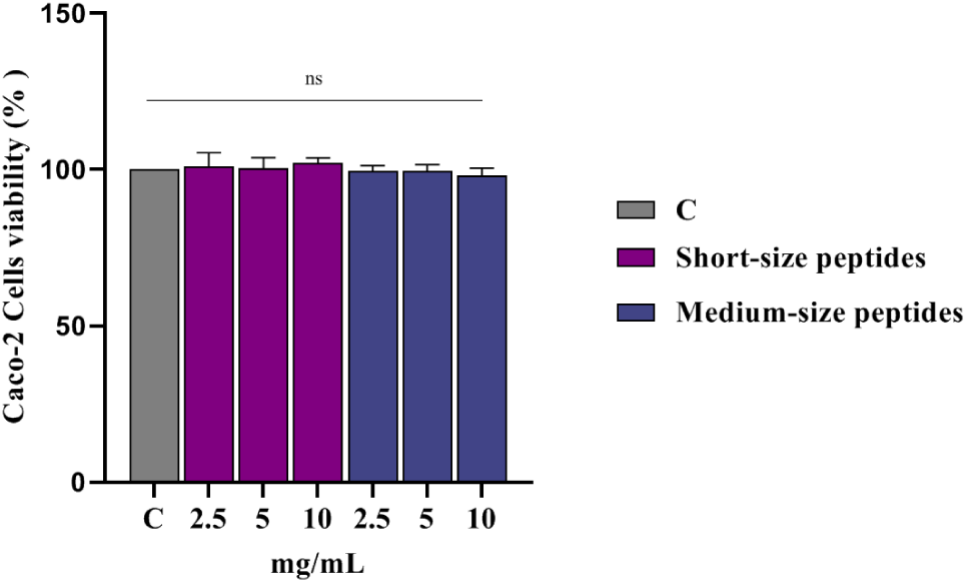
Effects of short- and medium-chain peptides on human intestinal Caco-2 cells (mean of six determinations performed in triplicate). C: control, ns: not significant.

These results align with studies on macroalgae extracts, such as ethanolic extracts of , , and , which also showed no significant cytotoxicity in Caco-2 cells under similar conditions.^36^ Furthermore, protein hydrolyzates have been confirmed as nontoxic at concentrations up to 2.5 mg/mL in Caco-2-derived lines, supporting the biocompatibility of red-algae-derived peptide mixtures.^37^

### 3.2 Direct ABTS Radical Scavenging Activity and FRAP Activity of Short- and Medium-Chain Peptides

The ABTS scavenging activity of the hydrolyzates was determined at 5.0, 10.0, and 20.0 mg/mL. As illustrated in Figure 2, short-chain peptides scavenged the ABTS radical by 24.9 ± 6.9%, 46.5 ± 5.95%, and 60,8 ± 0,7% at 5.0, 10.0, and 20.0 mg/mL, respectively. Medium-chain peptides lower the ABTS radical by 11,6 ± 2.5, 26.3 ± 7.2 and 36.1 ± 3.6% at the same range of concentration, respectively. Additionally, the FRAP assay measures the reduction of the ferric ion (Fe^3+^)-ligand complex to the intensely blue-colored ferrous (Fe^2+^) complex by antioxidants. The FRAP power of short- and medium-chain peptides was determined at concentrations of 1.0, 5.0, 10.0, and 20.0 mg/mL, respectively. Short hydrolyzate augmented the FRAP levels by 896.9 ± 152.1%, 3223.0 ± 1013.4%, 3780.6 ± 44.7%, and 4638.7 ± 87.8% at 1.0, 5.0, 10.0, and 20.0 mg/mL, respectively. On the other hand, medium hydrolyzate was able to enhance FRAP levels by 294.9 ± 31.5%, 1044.9 ± 102.9%, 1593.5 ± 57.2%, and 2180.6 ± 25.8% at the same assayed concentrations, respectively. Thus, short-chain peptide hydrolyzate was significantly more active in terms of direct antioxidant activity, as evidenced by its ability to reduce the ABTS radical and increase FRAP levels at all tested concentrations, compared to medium-chain peptide hydrolyzate. From a structural and physicochemical perspective, the antioxidant activity of peptides is modulated by several factors, including peptide chain length, amino acid composition, sequence, and the positioning of specific residues.^38^ Research suggests that short peptides often have greater antioxidant properties, mainly when they contain hydrophobic amino acids such as Leu or Val at the N-terminal or residues rich in sulfur as Cys, Met, containing aromatic residues (Phe, Trp, Tyr), or the imidazole group of His; these features enhance their ability to neutralize free radicals and reduce metal ions, which are key mechanisms underlying antioxidant activity.^39^ In this scenario, our results are in line with another study conducted on hempseed-derived peptides,^32^ where it has been demonstrated that short-chain (S) and medium-chain (M) peptides exhibit different antioxidant activities, being influenced by peptide chain length. In detail, the S peptides showed better antioxidant activity, particularly in the FRAP assay, S peptides resulted to be six times more active than M peptides, while in the DPPH assay, the S peptides were 1.6-fold more active than M peptides. Furthermore, previous studies have shown that short antioxidant peptides generally have a low molecular weight and a GRAVY (i.e., Grande Average of Hydropathicity index) score between −0.5 and +0.5, in addition to containing specific amino acids, differently from medium peptides, thus resulting in better antioxidant activity, as demonstrated for Soy Flour-Simulated Gastrointestinal Hydrolyzate.^40^ Consistent with these findings, our data also indicate that short peptides exhibit higher antioxidant activity when compared to medium-chain peptides.

**Figure 2:**
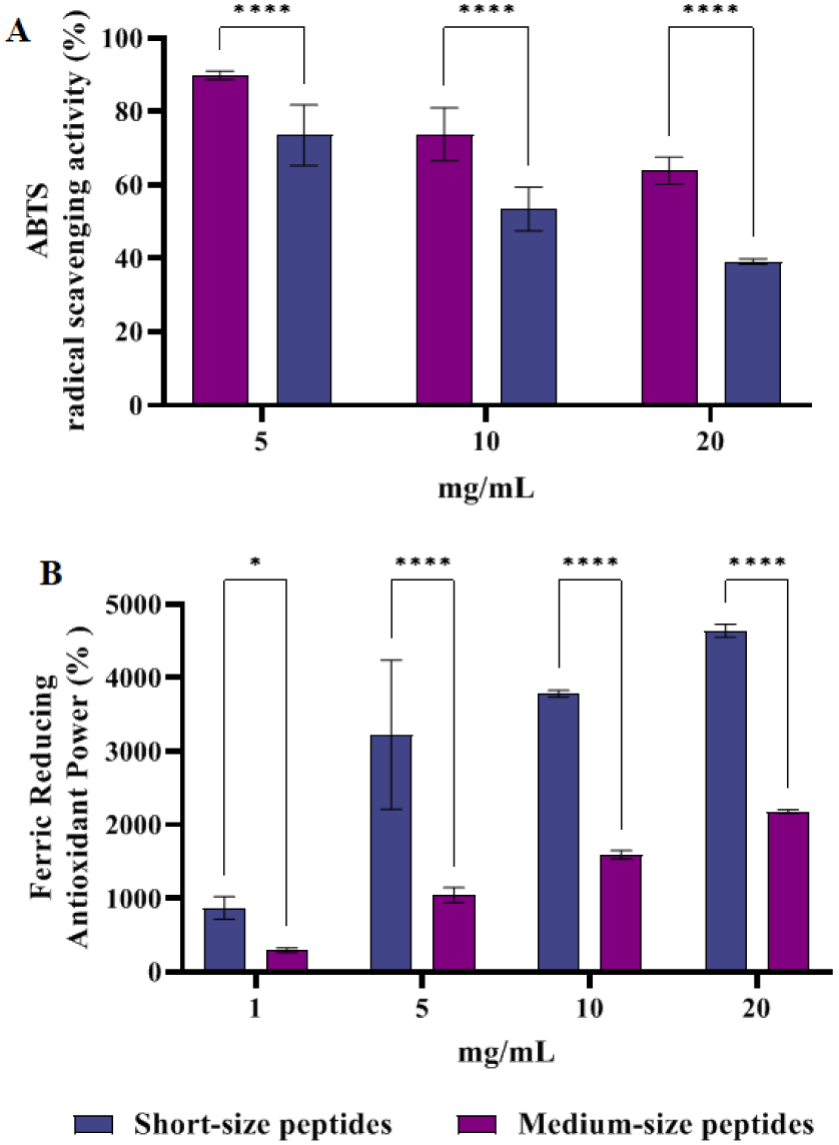
In vitro ABTS radical scavenging activity (A) and ferric-reducing antioxidant power (FRAP) activity (B) of short- and medium-chain peptides­(mean of six determinations performed in triplicate).

These results indicate that the short-chain peptide hydrolyzate exhibited significantly greater antioxidant capacity than the medium-chain fraction. Similar trends have been reported in previous studies on algae-derived and plant-based peptides. For instance, peptide hydrolyzates showed ABTS scavenging activity of approximately 40–50% at 10 mg/mL, which is comparable to the performance of our short-chain peptides.^41^ Moreover, peptides derived from displayed FRAP values near 1000% at 5 mg/mL,^42^ again confirming that our short peptide fraction is within or above the activity range reported for other marine macroalgae.

From a structural and physicochemical standpoint, the antioxidant capacity of peptides is determined by factors such as chain length, amino acid composition, and sequence, as well as the presence of specific residues. Short peptides frequently exhibit enhanced antioxidant activity when they contain hydrophobic amino acids (e.g., Leu, Val), sulfur-containing residues (Cys, Met), aromatic amino acids (Phe, Trp, Tyr), or the imidazole-containing residue His., all of which enhance their free radical scavenging and metal-ion reducing capacity. In this scenario, our findings align with literature on hempseed-derived peptides,^42^ where short-chain peptides exhibited up to six times higher FRAP activity than medium-chain peptides. Similarly, studies on soybean hydrolyzates demonstrated that short antioxidant peptides, typically having low molecular weight and GRAVY index between −0.5 and +0.5, were more effective due to their sequence composition.^43^ Consistent with these observations, our data confirm that short peptides from display higher antioxidant activity than their medium-chain counterparts.

### 3.3 Effect of the Short- and Medium-Chain Peptides on *In Vitro* DPP-IV Activity

Seaweeds are being increasingly exploited globally due to their dietary and nutritional benefits, particularly for their high content of amino acids and protein. Their hydrolyzates have been demonstrated to be enriched in bioactive peptides, possessing several biological properties, including antihypertensive and hypoglycemic effects.^44^ In line with our results, Dhaouafi and colleagues conducted an interesting study, characterizing novel bioactive peptides derived from Red Macroalgae using HPLC-MS/MS and identifying potential potent DPP-IV inhibitory peptides, which were quantified as comprising more than 90% of the peptides in the mixture.^45^ In addition, another study investigated the in vitro cardioprotective, antidiabetic, and antioxidant activity of protein hydrolyzates, a type of red seaweed found along the coasts of the North Atlantic, demonstrating that the peptide mixture had a DPP-IV inhibitory activity with IC50 values in the range 1.65–4.60 mg/mL, suggesting the use of macroalgae bioactive hydrolyzates as potential functional food ingredients, with good putative antidiabetic DPP-IV inhibitory activity.^46^ DPP-IV can be inhibited by various inhibitors that can access its active site located in a long cavity.^47^ DPP-IV inhibitory peptides are typically short sequences, with their inhibitory potency largely influenced by the position of specific residues, particularly at the N-terminus. High inhibitory activity is commonly observed in peptides containing Pro within the first to fourth N-terminal positions, and Gly, Ala, Phe, Val, or Leu at the C-terminus.^48^ Moreover, hydrophobic amino acids enhance substrate specificity and facilitate interaction with the enzyme’s functional hydrophobic pocket.^49^ Considering this evidence, the purified recombinant DPP-IV enzyme was used in *in vitro* assays to evaluate the capacity of short- and medium-chain peptides to inhibit the DPP-IV enzyme activity. Figure 3A demonstrates the dose-dependent decrease in vitro DPP-IV activity caused by both short- and medium-chain peptides evaluated at 1.0, 2.5, and 5.0 mg/mL. Short-chain peptides reduced DPP-IV activity by 13.8 ± 5.6%, 30.1 ± 6.7%, and 52.7 ± 6.8% at concentrations of 1.0, 2.5, and 5.0 mg/mL, respectively. In contrast, medium-chain peptides inhibited enzyme activity by 9.1 ± 2.4%, 23.6 ± 5.7%, and 68.2 ± 8.8%, respectively, at the same tested concentrations. Notably, medium-chain peptides could significantly inhibit DPP-IV activity at the highest tested concentration more efficiently than short-chain peptides. Additionally, DPP-IV is one of the well-known intestinal enzymes on the surface of Caco-2 cells and involved in food digestion.^50^ Consequently, this cell line was used to investigate the potential hypoglycemic action of hydrolyzates further. Results in Figure 3B reveal that short-chain peptides were able to inhibit DPP-IV activity by 5.2 ± 6.3%, 13.5 ± 4.4%, and 22.4 ± 6.8% at 2.5, 5.0, and 10.0 mg/mL, respectively, while medium-chain peptides inhibited the enzyme activity by 17.4 ± 0.9%, 26.7 ± 4.0%, and 32.9 ± 6.6%, at the same concentrations, respectively.

**Figure 3:**
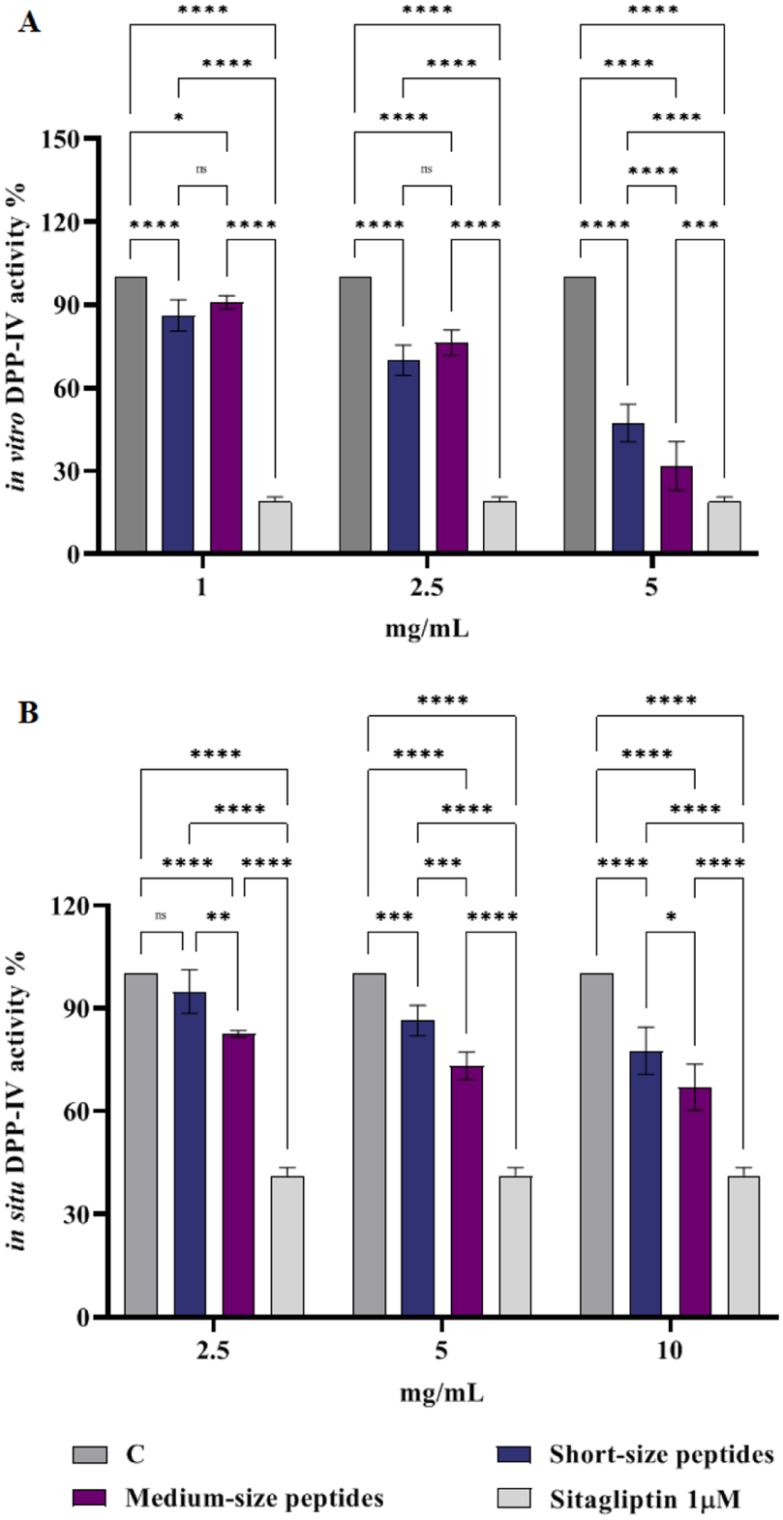
In vitro DPP-IV activity assay (A) and in situ DPP-IV activity evaluations on Caco-2 cells after 6 h of short- and medium-chain peptides treatments (B). Sitagliptin represents the positive control. Data are the means ± SD of three experiments performed in triplicate. C: control sample, ns: not significant. (*) *p* < 0.05, (**) *p* < 0.01, (***) *p* < 0.001, (****) *p* < 0.0001.

These findings are consistent with existing studies on red macroalgae-derived peptides. For example, protein hydrolyzates exhibited in vitro DPP-IV inhibitory activity with IC_50_ from 1.65 to 4.60 mg/mL, suggesting the potential use of such peptide mixtures as functional ingredients with hypoglycemic effects.^37^ Similarly, Dhaouafi et al. identified novel peptides from red macroalgae exhibiting DPP-IV inhibitory potential in over 90% of the sequences characterized by RP-HPLC-MS/MS, highlighting the high prevalence of bioactive motifs within this taxonomic group.^45^ In our study, although short-chain peptides effectively inhibited DPP-IV activity, medium-chain peptides demonstrated higher potency both *in vitro* and *in situ*, particularly at higher concentrations. This may reflect enhanced sequence compatibility of medium-chain peptides with the enzyme’s catalytic cleft, which is characterized by a deep and narrow binding pocket. The presence of key residues such as Pro, Val, Leu, or Ala at specific N- or C-terminal positions likely contributes to a more favorable interaction with the enzyme’s active site.^51^ Furthermore, while short-chain peptides showed higher hydrophobicity based on GRAVY index values, hydrophobicity alone is not sufficient to ensure DPP-IV inhibition. Rather, the synergistic effect of physicochemical properties—such as isoelectric point (pI), net charge at pH 8, hydrophobic moment, and peptide length—plays a central role in modulating inhibitory activity, as discussed in recent in silico and experimental studies.^52^ Taken together, these results suggest that the structural complexity of medium-chain peptides may afford a more effective inhibition of DPP-IV, reinforcing their potential as antidiabetic agents in functional food and nutraceutical formulations.

### 3.4 Effect of the Short- and Medium-Chain Peptides on *In Vitro* ACE Activity

Short- and medium-chain peptides were tested for *in vitro* ACE inhibition activity. The results are expressed as percentage ACE inhibition versus sample concentration (w/v) and are reported in Figure 4. Short- and medium-chain peptides showed ACE inhibitory activity at all the tested concentrations. Short-chain peptides inhibited ACE by 0.74 ± 0.12%, 1.81 ± 0.17%, 2.68 ± 0.21%, 8.46 ± 0.11%, 11.63 ± 0.24%, 15.21 ± 0.39%, and 19.53 ± 0.64% at 0.09, 0.17, 0.35, 0.69, 1.04, 1.38, and 2.07 mg/mL, respectively. Conversely, medium-chain peptides inhibited ACE by 0.30 ± 0.03%, 1.74 ± 0.04%, 2.41 ± 0.16%, 4.99 ± 0.19%, 8.05 ± 0.02%, 9.7 ± 0.38%, and 12.5 ± 0.42% at the same concentration range, respectively. Thus, short-chain peptides were more active than medium-chain ones at all the tested concentrations, showing more significant differences as concentration increased. Our results align with existing data on the ACE inhibitory activity of short- and medium-chain peptides, demonstrating consistency across various systems and applications.^53^ It is already established that the anti-ACE effect is strictly related to peptide length, with 2–8 residue peptides showing higher inhibitory activity. Our results align with previously published data regarding peptide mixtures obtained from hydrolyzed with Alcalase.^54^ However, within the family, happens to be the subtaxa displaying the lower ACE inhibitory activity as already demonstrated for , , and protein hydrolyzates within the family.^20,55,56^

**Figure 4:**
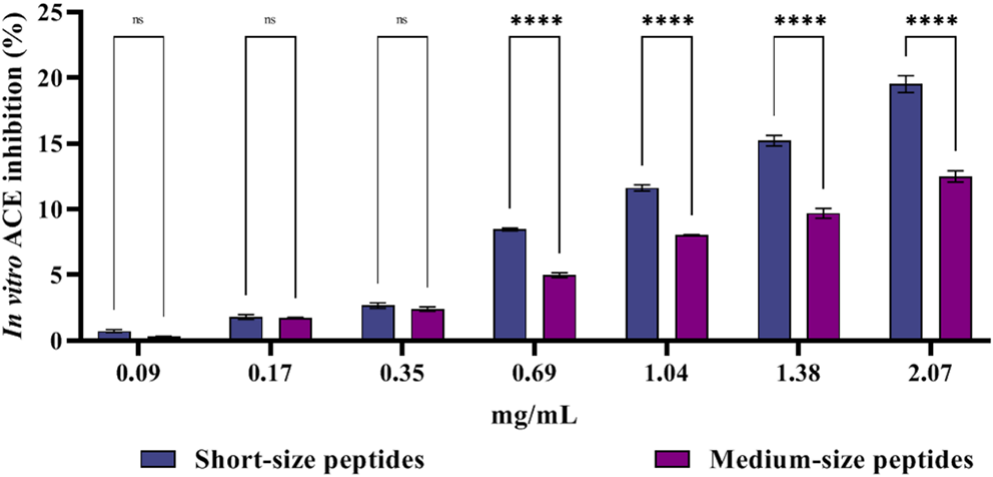
Percentage of *in vitro* ACE inhibition of short- and medium-chain peptides at different concentrations (0.09, 0.17, 0.35, 0.69, 1.04, 1.38, 2.07 mg/mL, mean of three determinations performed in duplicate).

These findings are consistent with previous reports showing that peptides with 2–8 amino acid residues generally exhibit stronger ACE inhibitory activity due to better access to the enzyme’s active site.^57^ Our results corroborate earlier studies on hydrolyzates generated with Alcalase, where short-chain fractions displayed superior ACE inhibition compared to longer peptides.^58^ Moreover, comparisons across members of the Gracilariaceae family reveal that typically exhibits lower ACE-inhibitory potential than related species like and .^20^

The superior ACE inhibition observed for short-chain peptides may be attributed to their smaller size and favorable amino acid composition, often including Pro, Leu, Ile, and aromatic residues like Tyr and Phe at C-terminal or N-terminal positions, which are recognized as critical for ACE binding affinity.^59^ In contrast, medium-chain peptides may suffer from steric hindrance or suboptimal orientation within the ACE catalytic cleft, limiting their effectiveness despite containing similar residues. Additionally, short-chain peptides often possess higher solubility and mobility in aqueous environments, enhancing their accessibility to enzyme targets. Overall, these data underscore the relevance of peptide size and structure in determining ACE inhibitory potential and support the preferential use of short-chain peptide fractions for the development of antihypertensive nutraceuticals.

## 4 Discussion

The study demonstrated that enzymatic hydrolysis of proteins, followed by molecular-weight-based fractionation, successfully yielded peptide mixtures with distinct biological profiles. Specifically, short-chain peptides exhibited higher antioxidant activity, as confirmed by ABTS and FRAP assays. In contrast, medium-chain peptides showed superior DPP-IV inhibitory activity, both in vitro and in Caco-2 cells. These effects can be attributed to the intrinsic characteristics of the peptides: shorter sequences often contain hydrophobic and aromatic residues that enhance radical scavenging and redox potential, whereas medium-length sequences may exhibit increased structural compatibility with the DPP-IV binding pocket. Furthermore, the ACE inhibitory activity was predominantly associated with short-chain peptides, aligning with the literature, which suggests that small peptides (2–8 residues) with hydrophobic or basic amino acids at the C-terminus exhibit a strong ACE-binding affinity. The differences observed in biological activity reflect not only peptide chain length but also their amino acid composition and physicochemical properties, as confirmed by GRAVY index analyses and peptidomic profiling. Finally, trans-epithelial transport experiments using Caco-2 cells demonstrated that both peptide fractions contain sequences capable of crossing the intestinal barrier, thereby reinforcing their potential for bioavailability and relevance in nutraceutical development.

In total, 97 medium-chain peptides were identified by database comparison against the UniProt protein sequence database for the *genus* (taxonomy ID 2774, comprising 3670 entries). Phycoerythrin is the primary phycobiliprotein in most red algae, as it is crucial for capturing light energy during photosynthesis.^60^ Studies have shown that these biological components are the primary source of bioactive peptides.^61,62^ Accordingly, the majority of identified medium-chain peptide sequences derived from α and β subunits of phycoerythrin as shown in Table S3. Interestingly, the peptide molecular weight distribution ranged from 599 to 1750 Da, with 55% being low molecular weight sequences (<1000 Da), which might result from extensive hydrolysis of the protein extract. Our findings align with those of other studies that produce low molecular weight peptides using Alcalase.^63^ Consistently, recent findings established that low molecular weight peptides from protein hydrolyzates can exhibit various bioactive effects.^64^ Short peptides were annotated using a specialized processing workflow on Compound Discoverer 3.1, as previously described.^26^ Overall, 362 short-chain peptides were putatively identified after manually interpreting the MS/MS spectra. Table S1 reports the identification data on the tentatively identified sequences. Since MS^3^ experiments are needed to distinguish leucine (Leu) and isoleucine (Ile), the three-letter nomenclature Xle and one-letter nomenclature J were employed to indicate either Leu or Ile. Ile and Leu were distinguished only when they appeared on the same side of the sequence, as the sequence containing Ile exhibited a shorter retention time due to the less polar nature of its R-chain compared to Leu. Literature evidence indicates that protein hydrolyzates from animal, plant, and marine sources possess in vitro DPP-IV inhibitory activity.^51,65^ In this context, protein hydrolyzates from red macroalgae have been recognized as a valuable source of peptides with inhibitory activity against DPP-IV and ACE. As previously noted, medium- and short-chain peptides derived from macroalgal proteins exhibit varying degrees of inhibition toward these enzymes. Bioactivity is linked to peptide hydrophobicity, enhancing the absorption rate and bioavailability. The hydrophobic character of these compounds also improves their ability to act as DPP-IV inhibitors.

Moreover, the presence of hydrophobic amino acids can enhance antioxidant activity by improving peptide solubility in nonpolar environments, thereby facilitating more effective interaction with and neutralization of free radicals.^66^ Thus, the *in silico* prediction of hydrophobic properties of identified medium and short-chain peptides was conducted using the GRAVY index (https://www.gravy-calculator.de). As is shown in Figure 5, the number of hydrophobic peptides was significantly higher for short-chain peptides than for medium-chain peptides. On the contrary, at the highest tested concentration, medium-chain peptides inhibited DPP-IV activity more efficiently than short-chain peptides. Hydrophobic or aromatic amino acids are commonly found in the N-terminal region of most DPP-IV inhibitors. However, many peptides lacking inhibitory activity also contain hydrophobic or aromatic residues at their N-terminus. This indicates that while these properties are beneficial for inhibition, they are insufficient to ensure inhibitory activity.^67^

**Figure 5:**
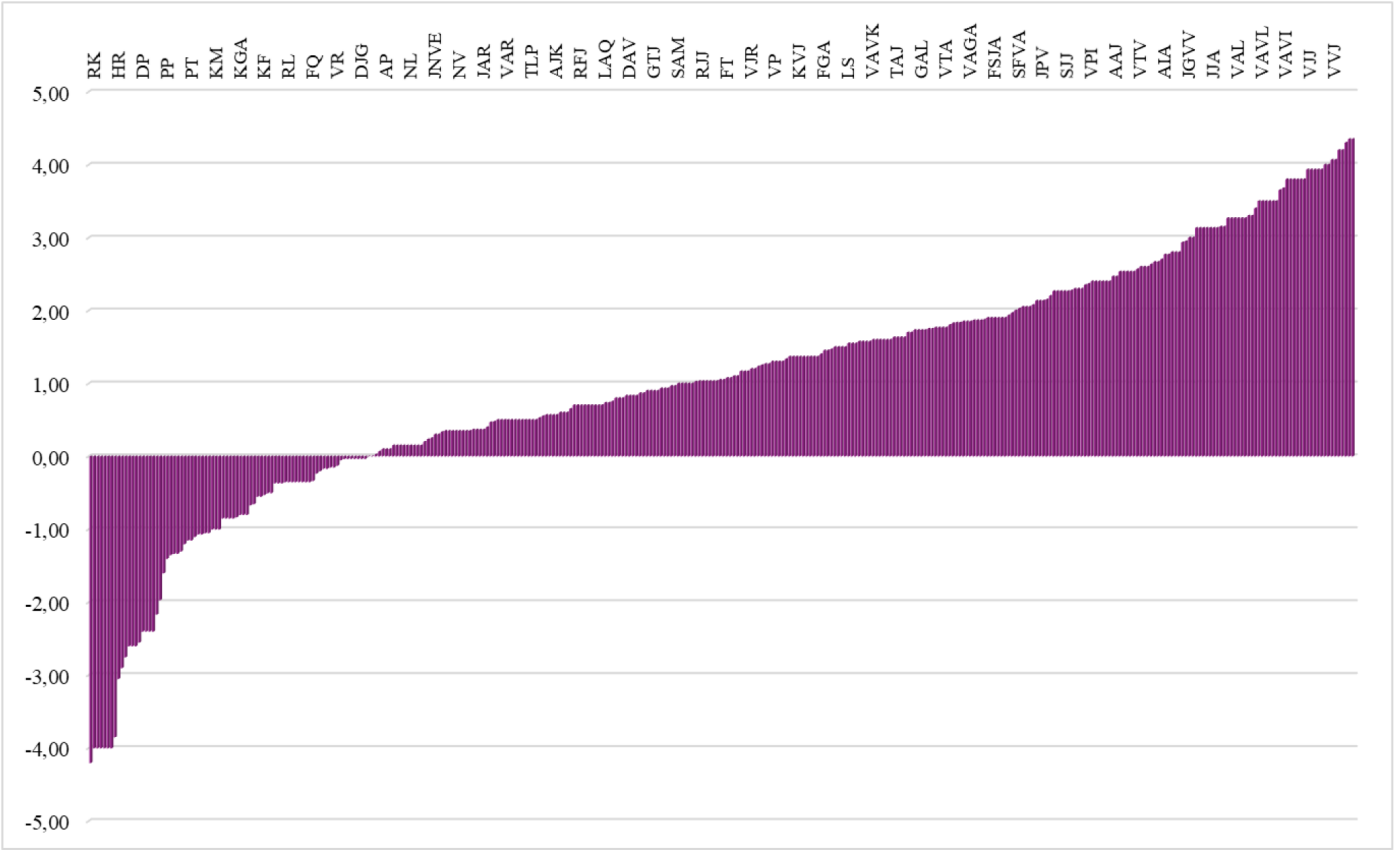
GRAVY index score (grand average of hydrophobicity) of putatively identified short-chain peptides. A score below 0 indicates hydrophilic properties, while a score above 0 indicates hydrophobic properties.

Bioactive peptides originating from food sources with ACE inhibitory properties represent a valuable resource for the discovery of novel antihypertensive therapeutics. Several studies have focused on elucidating their chemical characteristics and inhibitory mechanisms.^59,68^ Results imply that these peptides inhibited the enzyme by competing with the substrate to form the ACE-substrate complex, as reported in Table S1 short-chain peptides comprising aromatic residues (e.g., Phe, His, Trp, or Tyr) or Pro, Lys, Ile, Val, Leu, and Arg positioned at whether *C*- or *N*-terminus were among the most abundant peptides identified in this study. These results align with previous findings regarding key characteristics that contribute to ACE inhibition.^69^ Seaweeds offer a promising source of health-promoting bioactive peptides that may help prevent chronic diseases.^70^ Following ingestion, peptides must withstand enzymatic degradation, navigate the gastrointestinal tract, and cross the intestinal epithelium in an intact and bioactive form in order to reach target organs and exert their health-promoting effects. Nonetheless, the low metabolic stability and intestinal bioavailability of peptides remain significant challenges hindering their swift development as nutraceutical products.^71^ Caco-2 cells serve as a widely accepted in vitro model for studying the intestinal transport of food-derived bioactive peptides.Accordingly, Caco-2 cells were employed in this study to investigate the hydrolyzates’ inhibitory effects on key enzymes, including ACE and DPP-IV, as well as their antioxidant properties. Lastly, we investigated the intestinal *trans*-epithelial transport of short- and medium-chain peptides in differentiated human intestinal Caco-2 cell monolayers. Caco-2 cells have successfully differentiated and been incubated on their apical (AP) side with both short- and medium-chain peptide fractions at a 0.5 mg/mL concentration for 2 h. Following *trans*-epithelial transport experiments, both AP and BL solutions were collected and analyzed for each peptide fraction by UHPLC-HRMS and nanoHPLC-HRMS. Peptide bioavailability has been roughly estimated by evaluating the number of peptides crossing the barrier. Given the role of enzymatic processing in the digestive system, it is not unexpected to observe a small number of peptides in the BL solution compared to the starting hydrolyzate mixture, as peptides before crossing the membrane may be cleaved by brush-border peptidases, which actively break down larger peptides into smaller ones influencing absorption into the bloodstream, or they are uptaken by intracellular components of Caco-2 cells. Only sequences that remain undegraded when in contact with intestinal cells are more likely to pass through the barrier. Of the 362 short-chain peptides initially identified, 104 were present in the AP solution and 40 in the BL solution, unveiling that 38.5% of the short-chain peptides identified in the AP solution are transported across the intestinal membrane represented by the Caco-2 cell monolayer (Table S1). According to this hypothesis, our results show that 104 out of 362 peptides were found in the AP solution, suggesting that roughly 70% of the original short peptide fraction content might have been degraded by cellular brush border peptidases, resulting in significant alterations to the original peptidomic profile of the initial hydrolyzate mixture. Dipeptides are the most abundant moiety in the samples. Indeed, out of the 40 peptides identified in the BL solution, 19 are dipeptides, 16 are tripeptides, and 5 are tetrapeptides, mainly comprising hydrophobic or aromatic residues at their N-terminus. Peptide chain properties such as length, primary and secondary structures, and hydrophobicity influence their transport across the intestinal wall. After crossing the intestinal brush-border membrane, peptides can enter the bloodstream through four primary pathways: (i) carrier-mediated transport via PepT1, (ii) paracellular diffusion through tight junctions (TJs), (iii) transcytosis, and (iv) passive transcellular diffusion.^72^ PepT1 plays a significant role in peptide absorption; short peptides,^68^ such as dipeptides and tripeptides, are primarily transported via this route. In contrast, strongly hydrophobic peptides are typically absorbed through transcytosis or passive transcellular diffusion. The medium-chain peptide fraction was dominated by proteins that were consistently abundant across all conditions and mirrored the characteristic protein profile of red microalgae. Specifically, the most abundant protein was Phycoerythrin subunit A (Q6B8M6), a protein belonging to the phycobiliprotein class, which is typical of this species. Other abundant proteins in this class were detected (A0A6C0A9P9, A0A6C0AA91, A0A345U7P2). The second most abundant protein was Ribulose-1,5-bisphosphate carboxylase/oxygenase (RuBisCo, Q5UIT5, A0A0E3M4B8). Overall, other minor proteins were detected in the experiment, for a total of 37 proteins. Finally, we identified 75 peptides for the initial state (t_0_, AP at *t* = 0), 108 for the AP side of the membrane, and 65 for the BL side of the membrane (Table S2). In this case, the data suggested that several peptides (49% of the peptides) could interact with the cells and, indeed, reach the BL side of the cell layer.

Only 8% of peptides remained unchanged from the t_0_ state, as shown in Figure 6. Most of the peptides (32%) were shared in all three conditions, indicating that they were initially present in the tested mixture and could enter and cross the Caco-2 cells. Another significant part was exclusive to the apical side (32%), but not shared with the initial state, which indicated that these peptides could have been produced from the hydrolysis of precursor peptides, possibly due to peptidases located on the cell membrane, as already mentioned for the short-chain peptide fraction. Only a few peptides were exclusive to the basolateral side (8%). These results could be associated with a limited sensitivity of the analysis due to the low concentration of most peptides. The RSD deviation on peak areas of the matched peptides was thus considered to find statistically significant differences in the peptide distributions. Only at least a 2× concentration difference was considered, with RSD < 20%. From this analysis, three peptides (AVEGIARQPEVEGKIR, IEHTEDPHPR, SEGNKRL) were significantly more abundant on the basolateral side than the apical side, and the peptides were significantly more abundant on the basolateral side than in the t_0_ conditions (AVEGIARQPEVEGKIR, IEHTEDPHPR, AKLADNHDAVVK). Two of these peptides were shared in all three conditions but were significantly more abundant on the basolateral side, which could indicate good uptake of these sequences by Caco-2 cells. These results should be verified with absolute quantitative data to draw a reliable conclusion. Using a combination of analytical, biochemical, and cellular techniques, seaweed peptide fractions derived from the enzymatic digestion of protein extracts were evaluated for their antioxidant properties and their inhibitory effects on DPP-IV and ACE. The identification and bioactivity assessment of short- and medium-chain peptides derived from underscore the potential of this invasive red macroalga as a promising source for developing functional foods and nutraceuticals aimed at preventing and managing metabolic disorders, including hypertension and type 2 diabetes. The demonstrated ACE- and DPP-IV-inhibitory activities, combined with antioxidant effects and evidence of trans-epithelial transport in Caco-2 cells, highlight both the bioavailability and physiological relevance of these peptides. Collectively, these findings highlight the potential for translating the biotechnological valorization of infesting seaweed biomass into innovative health-promoting applications, aligning with the principles of the Sustainable Blue Economy and Circular Bioeconomy, and supporting future strategies in marine-derived preventive healthcare.

**Figure 6:**
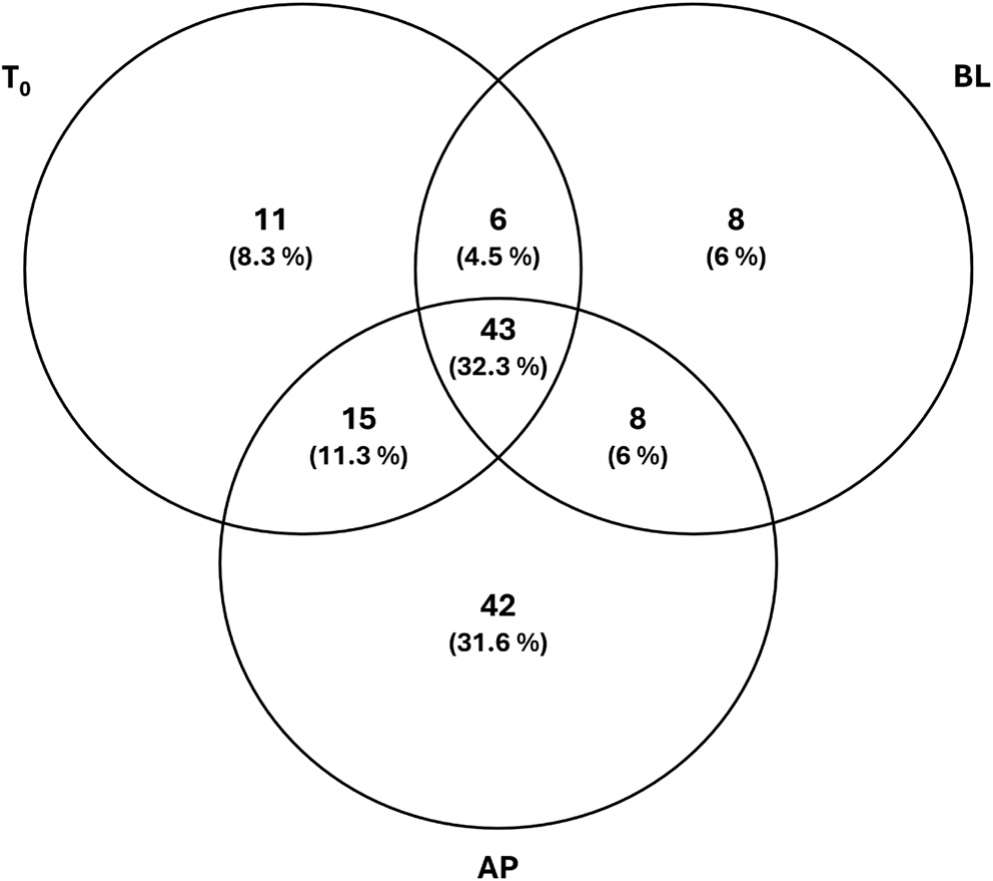
Distribution of the identified amino acid sequences among the three conditions (T0, AP, and BL) is reported in a Venn diagram.

This study highlights the potential of hydrolyzates from red macroalgae as a sustainable source of bioactive peptides for nutraceutical applications. In line with the principles of the Sustainable Blue Economy and the Bio-Based Circular Economy, underutilized seaweed species were strategically chosen and enzymatically hydrolyzed to promote their valorization within a circular and resource-efficient framework. The resulting peptide fractions were comprehensively characterized, and their biological activity was investigated through in vitro assays targeting key metabolic pathways. Specifically, both short- and medium-chain peptides exhibited inhibitory activity against DPP-IV and ACE, as well as significant antioxidant properties, as demonstrated by DPPH, FRAP, and ABTS assays. The short-chain peptide fraction showed a dose-dependent inhibition of DPP-IV activity in both acellular systems and Caco-2 intestinal epithelial cells. However, medium-chain peptides demonstrated superior inhibitory capacity *in situ* across all tested concentrations. Conversely, short-chain peptides were more effective in ACE inhibition, particularly at higher concentrations, indicating differentiated mechanisms of action depending on peptide length and target enzyme.

Importantly, trans-epithelial transport studies combined with peptidomics analysis confirmed the ability of several peptides to cross the intestinal barrier, suggesting favorable bioavailability profiles. Overall, these findings support the exploitation of red algae hydrolyzates as functional food ingredients or nutraceutical candidates aimed at preventing or managing chronic metabolic diseases, while contributing to the sustainable exploitation of marine bioresources. Further studies are warranted to evaluate the *in vivo* efficacy, safety, and bioavailability of the identified bioactive peptides. Elucidating their precise sequences and mechanisms of action will support targeted applications. In addition, investigating their stability during digestion and interaction with the gut microbiota could further enhance their nutraceutical potential.

## Supplementary Material







## Notes

Identification data for the short-chain peptides annotated from hydrolyzate (Table S1) (XLSX)
Identification data for the medium-chain peptides annotated from hydrolyzate (Table S2) (XLSX)
Identification data for the medium-chain peptides annotated from hydrolyzate after Caco-2 cells transepithelial transport assay (Table S3) (XLSX)
